# Functioning and well‐being in older children and adolescents with achondroplasia: A qualitative study

**DOI:** 10.1002/ajmg.a.62534

**Published:** 2021-10-13

**Authors:** Kathryn M. Pfeiffer, Meryl Brod, Alden Smith, Dorthe Viuff, Sho Ota, R. Will Charlton

**Affiliations:** ^1^ The Brod Group Mill Valley California USA; ^2^ Ascendis Pharma, Inc. Palo Alto California USA; ^3^ Ascendis Pharma, A/S Hellerup Denmark

**Keywords:** achondroplasia, adolescents, physical functioning, quality of life, school

## Abstract

The study aimed to explore how having achondroplasia affects older children and adolescents' day‐to‐day functioning and well‐being. Individual/focus group interviews were conducted with older children/adolescents between the ages of 9 to <18 years and diagnosed with achondroplasia to elicit key concepts. An adapted grounded theory approach informed the qualitative analysis of interview data. Thirty‐two children and adolescents completed interviews. Study results revealed five impact domains, including physical health, functioning, school impacts, emotional well‐being, and social well‐being. Frequently reported impacts on physical health included low stamina/tiring easily (81%) and back pain (69%). Key impacts in the functioning domain were difficulty with reaching objects or high places (84%) and walking long distances (75%). Emotional impacts included feeling different (63%), worried/scared (47%), and embarrassed/self‐conscious (47%). Impacts on social well‐being included difficulty with sports or physical play (81%) and others treating child as younger than their actual age (75%). The most frequent school impact was trouble participating in physical education (81%). A preliminary theoretical model depicting the experiences of older children/adolescents with achondroplasia was constructed based on the analysis. The preliminary theoretical model of older children and adolescents' experiences of living with achondroplasia may be used to inform future research and clinical practice.

## 1 INTRODUCTION

Achondroplasia, a common type of dwarfism, occurs due to a gain‐of‐function mutation in the *FGFR3* gene that leads to ligand‐independent signaling and ultimately affects bone and cartilage growth (He, Horton, & Hristova, 2010; Horton, Hall, & Hecht, 2007; Klag & Horton, 2016). The typical clinical characteristics of achondroplasia include short stature, disproportionally shortened arms and legs, macrocephaly, midface hypoplasia, small chest size, thoracolumbar kyphosis, lumbar hyperlordosis, limited elbow extension, shortened fingers with a trident configuration of the hands, joint hypermobility in hips/knees, and bowing of the legs (Pauli, 2019). Although there is no cure for achondroplasia, new treatments for children are currently being developed (Breinholt et al., 2019; Savarirayan et al., 2020).

The potential clinical complications associated with achondroplasia in children and adolescents are well known (Hunter, Bankier, Rogers, Sillence, & Scott Jr., 1998; Pauli, 2019). Chronic ear infections, sleep apnea, lumbar hyperlordosis, and overweight/obesity are frequent medical complications in children and adolescents with achondroplasia (Hunter et al., 1998; Wright & Irving, 2012). Children and adolescents with achondroplasia may also experience respiratory issues, conductive hearing loss, spinal stenosis/compression, back pain, and leg pain (Hunter et al., 1998; Wright & Irving, 2012). Clinical guidelines for managing achondroplasia in children and adolescents have been well established (Hoover‐Fong, Scott, Jones, & Committee on Genetics, 2020; Horton et al., 2007; Unger, Bonafe, & Gouze, 2017; Wright & Irving, 2012).

While the medical impacts associated with achondroplasia in older children and adolescents are well known, there is limited research on the nonmedical impacts of achondroplasia, including impacts on daily functioning and well‐being (Bloemeke, Sommer, Witt, Dabs, et al., 2019; Dogba, Rauch, Douglas, & Bedos, 2014; Gollust, Thompson, Gooding, & Biesecker, 2003; Sommer et al., 2017). Research has suggested that children and adolescents with achondroplasia may experience reduced quality of life compared to a reference population in four areas of functioning, including physical, emotional, social, and school (Witt et al., 2019). Additionally, older children and adolescents with achondroplasia have been shown to have greater stressors related to short stature compared to population averages (Nishimura & Hanaki, 2014). Other research, however, has found that health‐related quality of life in children, adolescents, and young adults with achondroplasia (patient‐reported) did not differ significantly from a normative reference population (Witt et al., 2017). The inconsistencies in studies of quality of life in children and adolescents with achondroplasia may be due in part to the use of inappropriate or generic measures not specific to achondroplasia (Bloemeke, Sommer, Witt, Bullinger, et al., 2019).

Currently, there is a lack of in‐depth, qualitative research focused on understanding children and adolescents' daily experiences with achondroplasia, which is necessary to develop a comprehensive and relevant measure of the impacts of achondroplasia on children and adolescents' quality of life. This study aimed to examine the impacts associated with achondroplasia on the functioning and well‐being of older children and adolescents. Additionally, the patient‐centered, qualitative data analysis was intended to inform the construction of a preliminary theoretical model illustrating how achondroplasia affects older children and adolescents' daily lives and well‐being.

## 2 METHODS

### 2.1 Editorial policies and ethical considerations

Prior to commencement, the study was approved by Copernicus Group Institutional Review Board (IRB), an independent IRB based in Research Triangle Park North Carolina, United States (US) (Protocol numbers TBG1‐18‐117 and 201905578). The research was conducted in accordance with the 1964 Helsinki Declaration and its later amendments or comparable ethical standards. Informed parental permission and child assent were obtained for all child/adolescent participants before starting the interviews.

### 2.2 Qualitative research study design and analysis

The qualitative study was designed in accordance with best research practices for patient‐reported health outcomes research (Lasch et al., 2010; Patrick et al., 2011a, 2011b; US Food and Drug Administration, Center for Drug Evaluation and Research (CDER), Center for Biologics Evaluation and Research (CBER), Center for Devices and Radiological Health (CDRH), 2009). A targeted literature review and expert interviews provided background information and clinical knowledge for the study. Concept elicitation (CE) interviews were then conducted with children and adolescents ages 9 to <18 years and diagnosed with achondroplasia to elicit their experiences and perspectives related to the condition.

The study followed an adapted grounded theory approach, in which a theory of patient experiences is developed based on the rigorous qualitative analysis of CE interview data, as well as clinical expertise and previous research (Brod, Tesler, & Christensen, 2009; Lasch et al., 2010). Based on the analysis of interview data, a preliminary theoretical model for the experiences of older children and adolescents diagnosed with achondroplasia was constructed.

#### 2.2.1 Concept elicitation

Individual CE interviews were conducted with experts to provide clinical and other relevant knowledge for understanding the experiences of children and adolescents with achondroplasia, including impacts on general health and well‐being. Individual expert telephone interviews were conducted with clinical experts in the US and Spain and with one leader of a US achondroplasia advocacy organization. Additional details regarding the expert interviews and the findings from these interviews have been reported elsewhere (Pfeiffer et al., 2021a).

Informed by the literature review and expert interviews, a semistructured interview guide was developed to question children and adolescents about their experiences associated with achondroplasia, including effects on physical health, day‐to‐day functioning, school participation, and general well‐being. To be eligible to participate in the interviews, children were required to be: (1) between the ages of 9 to <18 years of age at the time of interview; (2) diagnosed with achondroplasia; and (3) able to read, write, and speak English (in the US) or Spanish (in Spain). Exclusion criteria included having a cognitive impairment or other medical condition that would make it difficult for the child to take part in an individual/focus group interview about their experiences related to achondroplasia. It should be noted that the child/adolescent interviews were part of a larger study that also involved interviews with parents of children with achondroplasia. The study results based on parent interviews are reported in separate manuscripts (Pfeiffer et al., 2021a, 2021b).

Participants were recruited through advocacy organizations, clinician referrals, a professional market research organization, and “snowball” sampling. Details of recruitment strategies and recruiting targets have been described previously (Pfeiffer et al., 2021a). In an effort to capture a wide range of child/adolescent experiences, recruitment targets were set for country, child age, and whether child has a parent with achondroplasia. Brief telephone screening interviews with parents were used to verify child eligibility for the study. A modest honorarium was given to interview participants as a token of appreciation upon completion of the interview.

Individual CE interviews with children and adolescents were conducted by telephone and lasted approximately 60 min. One in‐person child/adolescent focus group interview, which lasted approximately 2 hr, was conducted in Spain. Child/adolescent interviews were conducted in English (US) or Spanish (Spain), and interviews were audio‐recorded and transcribed verbatim. A professional translation company was used to translate interviews from Spanish to English.

Guided by a grounded theory approach adapted for research in patient‐reported health outcomes, interview data were analyzed for conceptual themes (Lasch et al., 2010). An initial code list of concepts was constructed based on the semistructured CE interview guides. Interview transcripts were then coded through an iterative process in chronological order. Emerging concepts were added to the coding scheme, and transcripts previously coded were reviewed for the newly added concepts. Dedoose^©^, a web‐based application for qualitative and mixed methods research, was used for qualitative data analysis (SocioCultural Research Consultants, 2018).

To confirm that the interview sample size was adequate to capture all important themes, an analysis of thematic saturation was conducted for the child/adolescent interviewees in order of occurrence (Lasch et al., 2010).

#### 2.2.2 Preliminary theoretical model

To generate the preliminary theoretical model, a 2‐day meeting was held with the project team, composed of the Principal Investigator, the qualitative interviewers, and data analyst. Upon review of the analysis results, the team confirmed codes or revised codes when necessary. Following the review of the qualitative study, the team agreed on a set of criteria to identify impacts on child/adolescent well‐being as major or minor. Major impacts were intended to be important and relevant to children/adolescents with achondroplasia in different age groups, including ages 9 to <12 years, ages 12 to <15 years, and ages 15 to <18 years. The criteria that the team agreed upon for designating impacts as major were:

1. Endorsement of at least 30% of children/adolescents in each of the three child age groups (9 to <12 years; 12 to <15 years; and 15 to <18 years).
2. For child physical symptoms/health, the impact must not be a characteristic of the condition [e.g., small stature, shortening of arms and legs, macrocephaly, etc. (Pauli, 2019)].
3. Impacts were temporally proximal (rather than distal).

For the impacts that were not identified as major, the criteria for designating impacts as minor were:

1. Endorsement of at least 10% of children/adolescents in at least two of the three child age groups (9 to <12 years; 12 to <15 years; and 15 to <18 years).
2. Impacts were temporally proximal (rather than distal).

Following the designation of major and minor impacts, an initial theoretical model of the experiences of living with achondroplasia for older children and adolescents between the ages of 9 and <18 years was developed. The preliminary theoretical model aimed to illustrate impacts and impact domains and to distinguish between temporally proximal and more distal impacts. The theoretical model also identified factors that may modify the impacts associated with achondroplasia on children and adolescents' day‐to‐day lives and well‐being.

## 3 RESULTS

### 3.1 Concept elicitation interviews

#### 3.1.1 Sample description

Thirty‐two children and adolescents between the ages of 9 and <18 years and diagnosed with achondroplasia participated in individual interviews (*n* = 27) or a focus group interview (*n* = 5) in the US (*n* = 16) and Spain (*n* = 16). Parents of child/adolescent participants provided demographic information for their children. Average child age was 13 years (range, 9–17 years). Child/adolescent participants were divided into age groups for analysis purposes, including ages 9 to <12 years (41%, *n* = 13), ages 12 to <15 years (28%, *n* = 9), and ages 15 to <18 years (31%, *n* = 10). In total, 20 child participants were female (63%), and 12 child participants were male (38%). Racial/ethnic background of child was only asked in the US, and parents could report more than one category if applicable. Among US child participants, 6% identified as Asian‐American (*n* = 1), 13% identified as Black/African American (*n* = 2), 13% identified as Latino/Hispanic (*n* = 2), and 88% identified as White/Caucasian (*n* = 14). In Spain, 19% of child participants (*n* = 3) had a household income of <20,000€, 31% (*n* = 5) had an income of 20,001–40,000€, 19% (*n* = 3) had an income of 40,001–60,000€, 6% (*n* = 1) had an income of 60,001–80,000€, 6% (*n* = 1) had an income >100,000€, and 19% (*n* = 3) declined to answer. Among US child participants, 6% (*n* = 1) reported a household income of <$20,000, 6% (*n* = 1) had an income of $20,001–$40,000, 13% (*n* = 2) had an income of $80,001–$100,000, 63% (*n* = 10) had an income >$100,000, and 13% (*n* = 2) declined to report household income.

A majority of child/adolescent participants were diagnosed with achondroplasia in utero (53%, *n* = 17), while 31% (*n* = 10) were diagnosed at the time of birth, 13% (*n* = 4) were diagnosed between 2 and 6 months of age, and 3% (*n* = 1) had an unknown time/age of diagnosis due to adoption. Six child/adolescent participants (19%), all residing in the US, had one or both parents who also had achondroplasia. Parents also reported on children's general health status, which was described as “excellent” (25%, *n* = 8), “very good” (44%, *n* = 14), and “good” (31%, *n* = 10). No parent reported child's general health as “fair.” The average height of child participants was 118.2 cm (*SD*, 14.1; range, 94–149 cm). Average child weight was 34.2 kg (*SD*, 12.8), with a range of 20.4–68.0 kg. Five child participants (16%), all residing in Spain, previously had one or more elective limb lengthening surgeries.

#### 3.1.2 Child/adolescent concept elicitation interviews

A thematic saturation analysis of the 32 child/adolescent interviews confirmed that the interview sample size was adequate to capture a broad range of child/adolescent experiences and perspectives. In total, 108 concepts describing the physical signs/symptoms or complications related to achondroplasia (29 concepts) and the impacts associated with achondroplasia on children/adolescents and their families (79 concepts) were coded in the child and adolescent interview transcripts. After the 10th child/adolescent interview, 76% of concepts had been covered. Thematic saturation was reached at the 23rd child/adolescent interview, at which point 95% of concepts were discussed.

Children and adolescents described a range of physical symptoms and complications experienced due to achondroplasia. The most frequently discussed physical symptoms/complications of achondroplasia, not including clinical characteristics of achondroplasia (Pauli, 2019), are shown in Table 1. The most often mentioned physical symptom was pain (91%, *n* = 29), and the most common types of pain reported were back pain (69%, *n* = 22), joint pain (59%, *n* = 19), and leg pain (50%, *n* = 16). Children and adolescents also frequently discussed experiencing low stamina or tiring easily (81%, *n* = 26) and having teeth crowding or misalignment (69%, *n* = 22) due to achondroplasia. Other often mentioned physical symptoms/complications included ear infections or fluid in the ear (34%, *n* = 11), hearing problems or loss of hearing (34%, *n* = 11), and overweight/obesity (28%, *n* = 9).

**TABLE 1:** Most frequently reported physical symptoms/complications and impacts on daily functioning associated with achondroplasia

	Child age						Child total (*n* = 32)	
	9 to <12 years (*n* = 13)		12 to <15 years (*n* = 9)		15 to <18 years (*n* = 10)			
**Physical symptoms/complications (*n*, %)**								
Pain	12	92%	8	89%	9	90%	29	91%
Back pain	8	62%	7	78%	7	70%	22	69%
Joint pain	8	62%	7	78%	4	40%	19	59%
Leg pain	7	54%	3	33%	6	60%	16	50%
Low stamina/tiring easily	9	69%	8	89%	9	90%	26	81%
Teeth crowding/misalignment	7	54%	8	89%	7	70%	22	69%
Ear infections/fluid in ear	5	38%	3	33%	3	30%	11	34%
Hearing problems/loss	5	38%	2	22%	4	40%	11	34%
Overweight/obesity	0	0%	5	56%	4	40%	9	28%
**Impacts on daily functioning (*n*, %)**								
Use of adaptive devices (e.g., step stools)	13	100%	9	100%	10	100%	32	100%
Difficulty reaching objects/high places	11	85%	8	89%	8	80%	27	84%
Need assistance from others for tasks	9	69%	8	89%	9	90%	26	81%
Difficulty walking long distances	8	62%	7	78%	9	90%	24	75%
Issues with prolonged sitting or sitting without support	9	69%	4	44%	5	50%	18	56%
Difficulty being physically active	4	31%	5	56%	6	60%	15	47%
Difficulty running	10	77%	4	44%	0	0%	14	44%
Issues bathing/washing/grooming self	5	38%	2	22%	1	10%	8	25%
Difficulty with stairs or steps	1	8%	2	22%	4	40%	7	22%
Communication issues (e.g., due to trouble hearing)	2	15%	2	22%	3	30%	7	22%
Difficulty lifting/carrying objects	2	15%	3	33%	2	20%	7	22%
Challenges associated with travel	1	8%	2	22%	4	40%	7	22%
Difficulty hiking/climbing	3	23%	2	22%	2	20%	7	22%

*Note*: Clinical features of achondroplasia (e.g., short stature, disproportionate shortening of arms and legs, macrocephaly, hypermobility in joints, etc.) discussed by parents are not included in this table.

Children and adolescents also reported impacts associated with achondroplasia on their daily functioning (Table 1). The most often mentioned impacts on functioning included the use of adaptive devices (outside school; 100%, *n* = 32), difficulty reaching objects or high places (84%, *n* = 27), needing help from others for tasks (outside school; 81%, *n* = 26), difficulty walking long distances or for long periods of time (75%, *n* = 24), and difficulty sitting for long periods or sitting without back or leg support (56%, *n* = 18). Other impacts on daily functioning that children and adolescents frequently discussed included difficulty being physically active (47%, *n* = 15), difficulty running (44%, *n* = 14), issues with self‐care (e.g., bathing, washing, grooming; 25%, *n* = 8), difficulty with stairs or steps (22%, *n* = 7), communication issues (e.g., due to trouble hearing; 22%, *n* = 7), difficulty lifting or carrying objects (22%, *n* = 7), challenges associated with travel (22%, *n* = 7), and difficulty with hiking or climbing (22%, *n* = 7).

Children and adolescents also described emotional impacts that they experienced associated with achondroplasia (Table 2). The most frequently discussed emotional impacts were feeling different from others (63%, *n* = 20), feeling worried/scared (47%, *n* = 15), feeling embarrassed/self‐conscious (47%, *n* = 15), feeling frustrated/annoyed (41%, *n* = 13), and feeling sad/hurt (41%, *n* = 13). Children and adolescents also discussed feeling happy or experiencing joy in relation to achondroplasia (31%, *n* = 10), having a sense that life is difficult or challenging (25%, *n* = 8), feeling angry/mad (22%, *n* = 7), and feeling bothered (22%, *n* = 7).

**TABLE 2:** Most frequently reported impacts associated with achondroplasia on children/adolescents' emotional and social well‐being and school participation

	Child age						Child total (*n* = 32)	
	9 to <12 years (*n* = 13)		12 to <15 years (*n* = 9)		15 to <18 years (*n* = 10)			
**Impacts on emotional well‐being (*n*, %)**								
Feeling different	8	62%	7	78%	5	50%	20	63%
Worried/scared	5	38%	5	56%	5	50%	15	47%
Embarrassed/self‐conscious	4	31%	7	78%	4	40%	15	47%
Frustrated/annoyed	4	31%	3	33%	6	60%	13	41%
Sad/hurt	7	54%	3	33%	3	30%	13	41%
Feel happy/experience joy	2	15%	3	33%	5	50%	10	31%
Sense that life is difficult/challenging	1	8%	3	33%	4	40%	8	25%
Angry/mad	3	23%	3	33%	1	10%	7	22%
Bothered	1	8%	5	56%	1	10%	7	22%
**Impacts on social well‐being (*n*, %)**								
Difficulty participating in sports/physical play	10	77%	8	89%	8	80%	26	81%
Needing to explain achondroplasia to others	12	92%	6	67%	8	80%	26	81%
Being treated as younger than age	11	85%	5	56%	8	80%	24	75%
Issues participating in social activities	9	69%	8	89%	6	60%	23	72%
Teasing/bullying	11	85%	5	56%	5	50%	21	66%
Negative attention in public (e.g., staring, pointing)	7	54%	7	78%	6	60%	20	63%
Friendships/social activities through a community of people with dwarfism	5	38%	5	56%	6	60%	16	50%
Difficulty keeping up with other children their age physically	8	62%	3	33%	3	30%	14	44%
Being stigmatized	3	23%	6	67%	3	30%	12	38%
Peers treat differently	5	38%	0	0%	5	50%	10	31%
Positive impact on friendships	3	23%	5	56%	1	10%	9	28%
**Impacts on school participation (*n*, %)**								
Special adaptations/accommodations at school	12	92%	7	78%	9	90%	28	88%
Difficulty participating in physical education (P.E.)	12	92%	7	78%	7	70%	26	81%
Issues participating in class/schoolwork	9	69%	3	33%	7	70%	19	59%
Missed school days/time	4	31%	5	56%	8	80%	17	53%
Issues participating in school activities/field trips	4	31%	5	56%	4	40%	13	41%
Difficulty getting from place to place at school	3	23%	2	22%	8	80%	13	41%

In addition to impacts on emotional well‐being, children and adolescents expressed a range of impacts on their social well‐being associated with achondroplasia (Table 2). The most often reported impacts on children/adolescents' social well‐being were trouble participating in sports or other physical play (81%, *n* = 26), having to explain achondroplasia to others (81%, *n* = 26), others treating child as younger than his/her actual age (75%, *n* = 24), issues participating in social activities (72%, *n* = 23), being teased or bullied (66%, *n* = 21), and negative attention from others while in public spaces (e.g., staring, pointing; 63%, *n* = 20). Many children and adolescents discussed having friendships with other children diagnosed with achondroplasia/dwarfism and/or participating in social activities through a community of people with dwarfism, often facilitated by advocacy organizations (50%, *n* = 16). Other social impacts described included trouble keeping up physically with other children their age (44%, *n* = 14), being stigmatized by others (38%, *n* = 12), peers treating them differently (e.g., being protective of child, 31%, *n* = 10), and positive impacts on friendships (e.g., making friends easily, 28%, *n* = 9).

Children and adolescents also reported impacts associated with achondroplasia on school participation. The most often mentioned impacts on school participation included having special adaptations or accommodations at school (e.g., adapted chairs, more time to take tests, etc.; 88%, *n* = 28), difficulty participating in physical education (P.E.) class (81%, *n* = 26), issues participating in class or schoolwork (59%, *n* = 19), and missed school days or time (e.g., for doctor appointments; 53%, *n* = 17). Other school impacts discussed included issues participating in special school activities/events or field trips (41%, *n* = 13) and difficulty moving from one place to another at school (41%, *n* = 13).

### 3.2 Preliminary theoretical model

**FIGURE 1:**
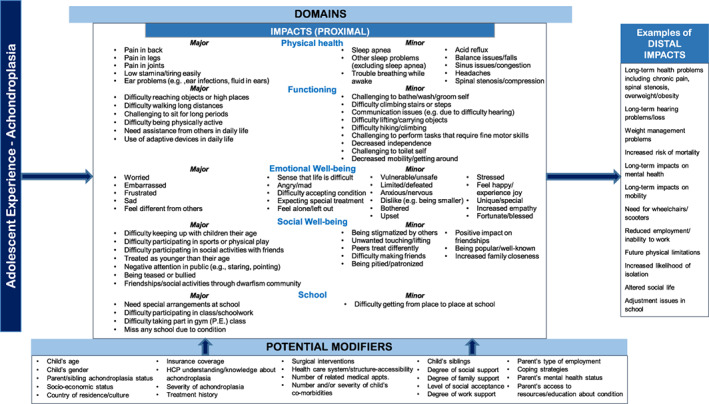
Preliminary theoretical model of adolescent experiences of living with achondroplasia (ages 9 to <18 years)

Informed by the analysis of child and adolescent interviews, an initial theoretical model for older children and adolescents' experiences of living with achondroplasia was developed for children/adolescents between the ages of 9 and <18 years (Figure 1). The preliminary theoretical model identified major and minor impacts associated with achondroplasia in the domains of physical health, functioning, emotional well‐being, social well‐being, and school. The preliminary model also distinguished between temporally proximal and more distal impacts. Potential modifiers to the impacts of achondroplasia were also noted, including child age, degree of family support, country of residence/culture, and health insurance coverage.

## 4 DISCUSSION

The study aimed to provide an in‐depth, qualitative analysis of the impacts associated with achondroplasia on adolescents' daily functioning and well‐being from the perspective of older children and adolescents with the condition. The findings demonstrate that older children and adolescents with achondroplasia experience a wide range of impacts in the domains of physical health, day‐to‐day functioning, emotional well‐being, social well‐being, and school participation. Reported impacts on older children and adolescents' physical symptoms and complications associated with achondroplasia are consistent with clinical knowledge (Horton et al., 2007; Pauli, 2019). It is notable that the majority of older children/adolescents (91%, *n* = 29) reported experiencing physical pain associated with achondroplasia, most often in the back (69%, *n* = 22), joints (59%, *n* = 19), and legs (50%, *n* = 16). Older children and adolescents also frequently discussed having low stamina/tiring easily and ear infections/fluid in ear related to achondroplasia. Compared to previous research on the physical symptoms/complications in younger children with achondroplasia based on parent reports, older children/adolescents more frequently mentioned experiencing teeth crowding/misalignment and overweight/obesity, but less frequently discussed having sleep apnea, respiratory issues, balance issues/falls, and speech issues (Pfeiffer et al., 2021a).

The study results are also in line with previous research suggesting that children ages 4–14 years with achondroplasia experience decreased quality of life, on average, compared to a reference population (Witt et al., 2019). The current study findings indicate that older children and adolescents face a number of challenges to functioning in daily life, including difficulty reaching objects and/or high places, trouble walking long distances, issues with prolonged sitting, and difficulty being physically active. All older children/adolescents reported the use of adaptive devices and most mentioned needing assistance from other people for daily tasks. Older children and adolescents less frequently mentioned difficulty with self‐care (e.g., bathing/washing/grooming, dressing/undressing, toileting self), trouble with stairs/steps, and difficulty with tasks requiring fine motor skills, which have been shown to be common issues for younger children with achondroplasia who often need caregiver assistance for self‐care and mobility (Ireland et al., 2011; Pfeiffer et al., 2021a, 2021b).

Frequently discussed emotional, social, and school impacts among older children and adolescents were generally similar to parent‐reported impacts in younger children aged 5 to <9 years with achondroplasia (Pfeiffer et al., 2021a). Common emotional impacts in older children and adolescents included feeling different, feeling worried/scared, and feeling embarrassed or self‐conscious in relation to achondroplasia. Some older children and adolescents also reported having happy or positive feelings related to achondroplasia. These findings should be considered in the context of previous research, which has suggested that adolescence is a period in which emotions, such as self‐consciousness and feeling worried, are heightened (Buchanan, Eccles, & Becker, 1992). The extent to which the emotional impacts observed in this study differ from typically developing adolescents is unclear and merits further research.

Frequently discussed social impacts included difficulty participating in sports or physical play, having to explain achondroplasia to others, others treating child as younger than actual age, experience of teasing/bullying, and negative attention in public, such as staring or pointing. Many older children and adolescents also described having friendships and participating in social activities/events through a community of people with dwarfism, often through achondroplasia/dwarfism advocacy organizations. Difficulty participating in gym/P.E. class was a frequently mentioned challenge to school participation. Most older children and adolescents reported having special adaptations or accommodations at school.

The qualitative findings also have implications for clinical practice. The study provides important data on the experiences and perspectives of older children and adolescents with achondroplasia, which may be a useful complement to established clinical guidelines for treating children and adolescents with achondroplasia (Hoover‐Fong et al., 2020; Trotter, Hall, & American Academy of Pediatrics Committee on Genetics, 2005; Wright & Irving, 2012). A better understanding of older children and adolescents' daily life experiences related to achondroplasia could improve clinician communication with older children/adolescents and their families and ultimately improve patient care. Evidence suggests that patient education and intervention programs tailored to address age‐specific impacts and issues associated with achondroplasia may also improve health‐related quality of life in children, adolescents, and young adults with achondroplasia (Witt et al., 2017). The preliminary theoretical model depicting the experiences of older children and adolescents with achondroplasia may be a helpful tool for clinicians, particularly for those who have limited experience caring for children and adolescents with achondroplasia.

Limitations of the study must be recognized in the interpretation of results. The findings were based on a nonrandom sample, which may not be entirely representative of older children and adolescents with achondroplasia. While multiple recruitment strategies were employed, a majority of respondents were recruited through achondroplasia advocacy organizations. Children and adolescents involved with achondroplasia/dwarfism advocacy organizations may have different experiences and perspectives compared to those who are not involved. Nevertheless, the CE interview sample was diverse in terms of participant background characteristics, and the analysis of thematic saturation indicated that the sample size was sufficient to capture the range of different experiences and perspectives among children and adolescents with achondroplasia. In addition, the study did not require clinical confirmation of a child's diagnosis with achondroplasia, so the possibility of a participant not having a diagnosis of achondroplasia cannot be ruled out with absolute certainty. This is unlikely, however, given that participants were recruited through advocacy organizations and clinicians experienced with achondroplasia and that all child/adolescent participants were able to discuss their experiences with achondroplasia at length during the interviews.

As the CE interviews were conducted in the US and Spain, some of the study findings may not be generalizable to other countries, which may have differing cultures or healthcare practices. Future research might explore the experiences of older children and adolescents with achondroplasia in other countries. A comprehensive and fully validated measure of child/adolescent quality of life that is specific to achondroplasia would be useful for cross‐cultural studies. Moreover, given the small sample size, statistical significance tests for group differences were not conducted, so observed differences in the study sample by age group or country may not be reflective of the population. Thus, any observed differences must be interpreted with caution, and additional research is needed to investigate possible differences in older children and adolescents' experiences across differing demographic groups.

It should also be noted that five of the participants in Spain had previously undergone elective limb‐lengthening surgeries, which are frequently done in adolescents with achondroplasia in Spain, but rarely in the US. Due to the small subsample of participants who had this surgery, an analysis of differences in impacts associated with achondroplasia between those who have and have not had limb lengthening surgery was not conducted. Research has found that children with achondroplasia who had lower limb lengthening surgery reported higher self‐esteem compared to those who did not have surgery, though there were no significant differences in general physical and mental health status (Kim, Balce, Agashe, Song, & Song, 2012). Limb lengthening surgery may have important implications for some of the impacts found in this study, such as difficulty reaching objects or high places, so further research is needed.

This study has shed light on the wide‐ranging experiences of older children and adolescents with achondroplasia, particularly the impacts associated with achondroplasia on older children and adolescents' day‐to‐day functioning and well‐being. The qualitative analysis identified major impacts associated with achondroplasia on older children and adolescents' physical health, daily functioning, emotional well‐being, social well‐being, and school life. The preliminary theoretical model of the experiences of older children/adolescents with achondroplasia may be a helpful guide for future research and clinical practice.

## CONFLICT OF INTEREST

K. M. Pfeiffer and M. Brod are consultants to the pharmaceutical industry, including Ascendis Pharma. A. Smith is an employee of Ascendis Pharma, Inc. D. Viuff is an employee of Ascendis Pharma, A/S. S. Ota and R. Will Charlton were employees of Ascendis Pharma, Inc. when the research was conducted.

## AUTHORS CONTRIBUTIONS

All authors participated in the conception and design of the study. K.M.P. and M.B. were involved in data collection, data analysis, interpretation of results, and drafting the manuscript. A.S., D.V., S.O., and R.W.C. contributed to critical manuscript revisions and intellectual content. All authors have given their approval of the final version of the manuscript and take responsibility for the manuscript's content and accuracy.

## Data Availability

The data for the research presented in the publication may be available on a case‐by‐case basis for reasonable requests from the corresponding author.

## References

[ajmga62534-bib-0001] Bloemeke, J. , Sommer, R. , Witt, S. , Bullinger, M. , Nordon, C. , Badia, F. J. , … Quitmann, J. (2019). Cross‐cultural selection and validation of instruments to assess patient‐reported outcomes in children and adolescents with achondroplasia. Quality of Life Research, 28(9), 2553–2563. 10.1007/s11136-019-02210-z 31093848

[ajmga62534-bib-0002] Bloemeke, J. , Sommer, R. , Witt, S. , Dabs, M. , Badia, F. J. , Bullinger, M. , & Quitmann, J. (2019). Piloting and psychometric properties of a patient‐reported outcome instrument for young people with achondroplasia based on the international classification of functioning disability and health: The Achondroplasia personal life experience scale (APLES). Disability and Rehabilitation, 41(15), 1815–1825. 10.1080/09638288.2018.1447028 29516753

[ajmga62534-bib-0003] Breinholt, V. M. , Rasmussen, C. E. , Mygind, P. H. , Kjelgaard‐Hansen, M. , Faltinger, F. , Bernhard, A. , … Hersel, U. (2019). TransCon CNP, a sustained‐release C‐type natriuretic peptide prodrug, a potentially safe and efficacious new therapeutic modality for the treatment of comorbidities associated with fibroblast growth factor receptor 3‐related skeletal dysplasias. The Journal of Pharmacology and Experimental Therapeutics, 370(3), 459–471. 10.1124/jpet.119.258251 31235532

[ajmga62534-bib-0004] Brod, M. , Tesler, L. E. , & Christensen, T. L. (2009). Qualitative research and content validity: Developing best practices based on science and experience. Quality of Life Research, 18(9), 1263–1278. 10.1007/s11136-009-9540-9 19784865

[ajmga62534-bib-0005] Buchanan, C. M. , Eccles, J. S. , & Becker, J. B. (1992). Are adolescents the victims of raging hormones: Evidence for activational effects of hormones on moods and behavior at adolescence. Psychological Bulletin, 111(1), 62–107. 10.1037/0033-2909.111.1.62 1539089

[ajmga62534-bib-0007] Dogba, M. J. , Rauch, F. , Douglas, E. , & Bedos, C. (2014). Impact of three genetic musculoskeletal diseases: A comparative synthesis of achondroplasia, Duchenne muscular dystrophy and osteogenesis imperfecta. Health and Quality of Life Outcomes, 12, 151. 10.1186/s12955-014-0151-y 25649344 PMC4332447

[ajmga62534-bib-0008] Gollust, S. E. , Thompson, R. E. , Gooding, H. C. , & Biesecker, B. B. (2003). Living with achondroplasia in an average‐sized world: An assessment of quality of life. American Journal of Medical Genetics. Part A, 120A(4), 447–458. 10.1002/ajmg.a.20127 12884421

[ajmga62534-bib-0009] He, L. , Horton, W. , & Hristova, K. (2010). Physical basis behind achondroplasia, the most common form of human dwarfism. The Journal of Biological Chemistry, 285(39), 30103–30114. 10.1074/jbc.M109.094086 20624921 PMC2943285

[ajmga62534-bib-0010] Hoover‐Fong, J. , Scott, C. I. , Jones, M. C. , & Committee on Genetics . (2020). Health supervision for people with achondroplasia. Pediatrics, 145(6), e20201010. 10.1542/peds.2020-1010 32457214

[ajmga62534-bib-0011] Horton, W. A. , Hall, J. G. , & Hecht, J. T. (2007). Achondroplasia. Lancet, 370(9582), 162–172. 10.1016/S0140-6736(07)61090-3 17630040

[ajmga62534-bib-0012] Hunter, A. G. , Bankier, A. , Rogers, J. G. , Sillence, D. , & Scott, C. I., Jr. (1998). Medical complications of achondroplasia: A multicentre patient review. Journal of Medical Genetics, 35(9), 705–712. 10.1136/jmg.35.9.705 9733026 PMC1051420

[ajmga62534-bib-0013] Ireland, P. J. , McGill, J. , Zankl, A. , Ware, R. S. , Pacey, V. , Ault, J. , … Johnston, L. M. (2011). Functional performance in young Australian children with achondroplasia. Developmental Medicine and Child Neurology, 53(10), 944–950. 10.1111/j.1469-8749.2011.04050.x 21838822

[ajmga62534-bib-0015] Kim, S. J. , Balce, G. C. , Agashe, M. V. , Song, S. H. , & Song, H. R. (2012). Is bilateral lower limb lengthening appropriate for achondroplasia?: Midterm analysis of the complications and quality of life. Clinical Orthopaedics and Related Research, 470(2), 616–621. 10.1007/s11999-011-1983-y 21785895 PMC3254769

[ajmga62534-bib-0016] Klag, K. A. , & Horton, W. A. (2016). Advances in treatment of achondroplasia and osteoarthritis. Human Molecular Genetics, 25(R1), R2–R8. 10.1093/hmg/ddv419 26443596

[ajmga62534-bib-0017] Lasch, K. E. , Marquis, P. , Vigneux, M. , Abetz, L. , Arnould, B. , Bayliss, M. , … Rosa, K. (2010). PRO development: Rigorous qualitative research as the crucial foundation. Quality of Life Research, 19(8), 1087–1096. 10.1007/s11136-010-9677-6 20512662 PMC2940042

[ajmga62534-bib-0018] Nishimura, N. , & Hanaki, K. (2014). Psychosocial profiles of children with achondroplasia in terms of their short stature‐related stress: A nationwide survey in Japan. Journal of Clinical Nursing, 23(21–22), 3045–3056. 10.1111/jocn.12531 25453127

[ajmga62534-bib-0019] Patrick, D. L. , Burke, L. B. , Gwaltney, C. J. , Leidy, N. K. , Martin, M. L. , Molsen, E. , & Ring, L. (2011a). Content validity—establishing and reporting the evidence in newly developed patient‐reported outcomes (PRO) instruments for medical product evaluation: ISPOR PRO good research practices task force report: Part 1—eliciting concepts for a new PRO instrument. Value in Health, 14(8), 967–977. 10.1016/j.jval.2011.06.014 22152165

[ajmga62534-bib-0020] Patrick, D. L. , Burke, L. B. , Gwaltney, C. J. , Leidy, N. K. , Martin, M. L. , Molsen, E. , & Ring, L. (2011b). Content validity—establishing and reporting the evidence in newly developed patient‐reported outcomes (PRO) instruments for medical product evaluation: ISPOR PRO good research practices task force report: Part 2—assessing respondent understanding. Value in Health, 14(8), 978–988. 10.1016/j.jval.2011.06.013 22152166

[ajmga62534-bib-0021] Pauli, R. M. (2019). Achondroplasia: A comprehensive clinical review. Orphanet Journal of Rare Diseases, 14(1), 1. 10.1186/s13023-018-0972-6 30606190 PMC6318916

[ajmga62534-bib-0022] Pfeiffer, K. M. , Brod, M. , Smith, A. , Gianettoni, J. , Viuff, D. , Ota, S. , & Charlton, R. W. (2021a). Assessing physical symptoms, daily functioning, and well‐being in children with achondroplasia. American Journal of Medical Genetics, Part A, 185(1), 33–45. 10.1002/ajmg.a.61903 33084192 PMC7756853

[ajmga62534-bib-0023] Pfeiffer, K. M. , Brod, M. , Smith, A. , Gianettoni, J. , Viuff, D. , Ota, S. , & Charlton, R. W. (2021b). Assessing the impacts of having a child with achondroplasia on parent well‐being. Quality of Life Research, 30(1), 203–215. 10.1007/s11136-020-02594-3 32803627 PMC7847864

[ajmga62534-bib-0024] Savarirayan, R. , Tofts, L. , Irving, M. , Wilcox, W. , Bacino, C. A. , Hoover‐Fong, J. , … Day, J. (2020). Once‐daily, subcutaneous vosoritide therapy in children with achondroplasia: A randomised, double‐blind, phase 3, placebo‐controlled, multicentre trial. Lancet, 396(10252), 684–692. 10.1016/S0140-6736(20)31541-5 32891212

[ajmga62534-bib-0006] SocioCultural Research Consultants (2018). Dedoose (Version 8.0.35), web application for managing, analyzing, and presenting qualitative and mixed method research data [Software]. Los Angeles, CA: SocioCultural Research Consultants, LLC. Available from: https://www.dedoose.com

[ajmga62534-bib-0025] Sommer, R. , Blomeke, J. , Dabs, M. , Witt, S. , Bullinger, M. , & Quitmann, J. (2017). An ICF‐CY‐based approach to assessing self‐ and observer‐reported functioning in young persons with achondroplasia ‐ development of the pilot version of the Achondroplasia Personal Life Experience Scale (APLES). Disability and Rehabilitation, 39(24), 2499–2503. 10.1080/09638288.2016.1226969 27636099

[ajmga62534-bib-0026] Trotter, T. L. , Hall, J. G. , & American Academy of Pediatrics Committee on Genetics . (2005). Health supervision for children with achondroplasia. Pediatrics, 116(3), 771–783. 10.1542/peds.2005-1440 16140722

[ajmga62534-bib-0027] Unger, S. , Bonafe, L. , & Gouze, E. (2017). Current care and investigational therapies in achondroplasia. Current Osteoporosis Reports, 15(2), 53–60. 10.1007/s11914-017-0347-2 28224446 PMC5435778

[ajmga62534-bib-0028] US Food and Drug Administration, Center for Drug Evaluation and Research (CDER), Center for Biologics Evaluation and Research (CBER), Center for Devices and Radiological Health (CDRH) . (2009). Guidance for industry patient‐reported outcome measures: use in medical product development to support labeling claims. Rockville, MD: FDA. December 2009. Retrieved from https://www.fda.gov/regulatory‐information/search‐fda‐guidance‐documents/patient‐reported‐outcome‐measures‐use‐medical‐product‐development‐support‐labeling‐claims.

[ajmga62534-bib-0029] Witt, S. , Kolb, B. , Bloemeke, J. , Mohnike, K. , Bullinger, M. , & Quitmann, J. (2019). Quality of life of children with achondroplasia and their parents ‐ a German cross‐sectional study. Orphanet Journal of Rare Diseases, 14(1), 194. 10.1186/s13023-019-1171-9 31399110 PMC6688231

[ajmga62534-bib-0030] Witt, S. , Rohenkohl, A. , Bullinger, M. , Sommer, R. , Kahrs, S. , Klingebiel, K. H. , … Quitmann, J. (2017). Understanding, assessing and improving health‐related quality of life of young people with achondroplasia ‐ a collaboration between a patient organization and academic medicine. Pediatric Endocrinology Reviews, 15(Suppl 1), 109–118. 10.17458/per.vol15.2017.wrm.improvinghealthrelatedquality 29292874

[ajmga62534-bib-0031] Wright, M. J. , & Irving, M. D. (2012). Clinical management of achondroplasia. Archives of Disease in Childhood, 97(2), 129–134. 10.1136/adc.2010.189092 21460402

